# Socioeconomic differences in help-seeking intention for stress urinary incontinence: evidence from an information–motivation–behavioral skills model framework

**DOI:** 10.3389/fpubh.2026.1794464

**Published:** 2026-04-29

**Authors:** Xin Ge, Zizhen Dai, Yujie Liu, Shunyu Tao, Birong Wu, Yong Cai, Jin Qiu

**Affiliations:** 1 Institute of Clinical Research, Tongren Hospital, Shanghai Jiao Tong University School of Medicine, Shanghai, China; 2 Department of Obstetrics and Gynecology, Tongren Hospital, Shanghai Jiao Tong University School of Medicine, Shanghai, China

**Keywords:** stress urinary incontinence, help-seeking intention, subjective norms, socioeconomic status, Information–Motivation–Behavioral Skills model, older women

## Abstract

**Background:**

Stress urinary incontinence is common among older women and can substantially affect quality of life, yet medical help-seeking remains low. While socioeconomic disparities have been associated with variations in health behavior, the underlying psychosocial pathways through which these disparities manifest are not well understood. This study aimed to identify psychosocial factors that influence intention to seek care for urinary incontinence and to assess whether they explain the association between socioeconomic status and help-seeking intention.

**Methods:**

A cross-sectional survey was conducted among women aged ≥ 50 years with questionnaire-identified stress urinary incontinence recruited from 10 community health centers in Shanghai (*N* = 585). Measures were developed under the IMB model. Socioeconomic status was derived using latent class analysis. The primary outcome was intention to seek medical care within six months, assessed on a five-point scale. Ordered logistic regression models were used to examine associations and to evaluate attenuation of the socioeconomic effect after inclusion of model constructs.

**Results:**

Only 38.1% of participants reported high help-seeking intention (≥4 on a 1–5 scale). Subjective norms (cOR = 2.81, 95% CI 2.25–3.51) and stigma (cOR = 1.80, 95% CI 1.35–2.40) were the strongest correlates of higher intention, followed by patient–provider communication (cOR = 1.27, 95% CI 1.04–1.54). Knowledge was negatively associated with intention (cOR = 0.81, 95% CI 0.67–0.97). After sequentially adding information, motivational, and behavioral factors from the IMB framework, the association between socioeconomic status and help-seeking intention was attenuated by approximately 80% and was no longer statistically meaningful.

**Conclusions:**

Social and psychological factors, particularly subjective norms, play a central role in shaping intention to seek care for stress urinary incontinence among older women. These factors substantially explain differences originally attributed to socioeconomic position. Interventions that shift community norms, reduce stigma, and strengthen communication in primary care may improve engagement with treatment and reduce unmet needs in aging populations.

## 1 Background

Rapid population aging is reshaping health needs globally and amplifying the burden of chronic and geriatric conditions (1). Stress urinary incontinence (SUI)—involuntary leakage with coughing, sneezing, or exertion—is common and persistently under-treated among older women (2). Beyond physical symptoms, SUI is associated with diminished health-related quality of life, elevated risks of depression and social withdrawal, and non-trivial societal costs (3, 4). In China, adults aged ≥ 60 years already comprise more than 15.6 % of the population, underscoring the urgency of addressing age-related health needs (4–6). Although effective treatments such as pelvic-floor muscle training and surgical procedures are available, only 7–10.7 % of women with incontinence seek professional care (7–9).

A growing literature identifies multiple psychosocial and structural barriers to care-seeking for SUI. Shame, embarrassment, and the perception that leakage is a normal part of aging often discourage disclosure (10). Knowledge deficits and low self-efficacy to communicate symptoms with clinicians further reduce treatment (11, 12). Socioeconomic status (SES)—a fundamental cause of health inequality—shapes access to information, resources, and perceived control, thereby influencing help-seeking behavior (13, 14). However, evidence remains fragmented regarding how SES interacts with psychosocial mechanisms to produce disparities in care-seeking for stigmatized conditions such as SUI.

Social environments are particularly salient in this context. Research on older Asian populations suggests that social norms and cohesion exert paradoxical effects: they may offer mutual trust and emotional support, yet can also heighten reputation concerns that deter help-seeking (13). In closely knit urban communities in China, older adults' social networks are often family-centered and geographically bounded (15, 16). Within these interdependent networks, family members and neighbors act as moral gatekeepers. Their approval can legitimize medical help-seeking, whereas disapproval or anticipated gossip imposes psychological and reputational costs (17, 18). Such pressures are often stronger among lower-SES groups, whose local ties are dense but externally constrained, limiting perceived autonomy and increasing the salience of social expectations (19, 20). These dynamics echo findings from Japan, where strong community cohesion was linked to greater reputation concern and reluctance to seek help among less-educated older adults.

The Information–Motivation–Behavioral Skills (IMB) model provides a useful framework for integrating these multilevel influences. It posits that health-related behaviors depend on sufficient information, personal and social motivation, and behavioral skills (21, 22). In the SUI context, knowledge represents information; stigma and life burden capture personal motivation; subjective norms and patient–provider communication reflect social motivation; and care-seeking self-efficacy represents behavioral skills. While SES determines access to informational and psychosocial resources, the IMB model conceptualizes these constructs as proximal and potentially modifiable determinants of intention and behavior. Despite its theoretical promise, this framework has rarely been applied to urinary incontinence or to aging populations in collectivist settings (21).

**Figure 1:**
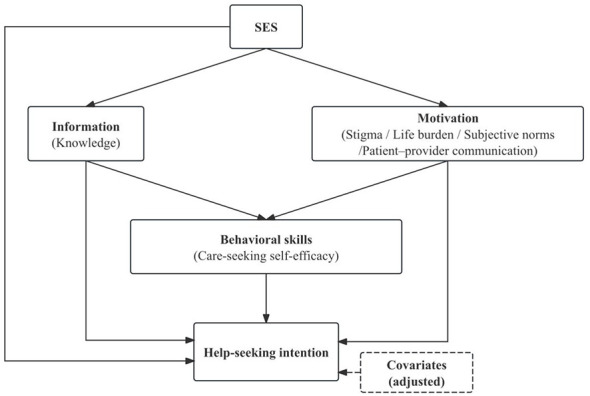
Conceptual framework of help-seeking intention based on the IMB model.

Guided by the IMB framework (Figure 1), this study investigates how informational, motivational, and behavioral factors shape help-seeking intention for SUI among community-dwelling women aged ≥ 50 years in urban China, and how these psychosocial determinants relate to socioeconomic context. Specifically, we aim to identify which proximal factors—particularly subjective norms, stigma, and self-efficacy—are most strongly associated with help-seeking intention, and to evaluate whether social motivation outweighs socioeconomic status in explaining variation across individuals. The findings are expected to clarify modifiable levers for interventions that normalize discussion of SUI and promote timely engagement with care in China's aging urban communities.

## 2 Materials and methods

### 2.1 Study design and instrument

We conducted a cross-sectional study in Shanghai, China, from June to August 2024. A self-administered questionnaire was developed under the IMB framework, informed by literature review and expert consultation. Instrument development followed a multi-step process: (1) item generation and mapping to IMB constructs; (2) expert review for content relevance and clarity; (3) cognitive interviews with target respondents to assess comprehension and flow; and (4) a pilot test to refine wording, skip patterns, and response scales. Revisions were implemented to enhance face and content validity and to minimize respondent burden.

### 2.2 Setting, recruitment, and ethics

Participants were recruited at 10 Community Health Service Centers in Changning District, Shanghai, using onsite convenience sampling. Clinic staff introduced the study to potentially eligible women in waiting areas and during community outreach; trained research assistants then screened for eligibility and invited participation. Written informed consent was obtained prior to survey initiation. Participation was voluntary with the option to withdraw at any time, and personal identifiers were not retained in the analytical dataset. To protect privacy and encourage disclosure regarding urinary symptoms, female researchers were arranged to assist with the completion of the questionnaire survey.

### 2.3 Data collection platform and quality control

The final questionnaire was administered online via Wenjuanxing (https://www.wjx.cn/), a widely used Chinese platform for questionnaires. The survey implemented front-end controls (mandatory responses for key variables; real-time logic and range checks) and IP-/device-based restrictions to limit duplicate submissions. Respondents completed the survey using center-provided tablets or personal smartphones by scanning a QR code; trained staff provided technical support without influencing responses. For older participants who required assistance, staff offered read-aloud and navigation help while avoiding any guidance on response content.

### 2.4 Eligibility criteria

Inclusion criteria were: (1) women aged ≥50 years; (2) self-reported SUI identified using the International Consultation on Incontinence Questionnaire urinary incontinence Short Form (ICIQ-UI) with leakage predominantly associated with physical activity, coughing, or sneezing; (3) residence in the community for ≥6 months; and (4) provision of written informed consent.

Exclusion criteria included: (1) Isolated urge urinary incontinence without stress-related symptoms; (2) history of pelvic floor surgery (e.g., pelvic reconstructive surgery) or currently receiving treatment for SUI; (3) Life-threatening cardiovascular or cerebrovascular diseases, or malignant tumors; (4) Cognitive or psychiatric disorders that hinder questionnaire completion; (5) responses below the 5th percentile of survey duration; (6) Refusal to participate in the study.

### 2.5 Sample size and recruitment flow

Sample size calculations were based on detecting an overall association between SES and the five-level help-seeking intention in an ordinal logistic regression framework while adjusting for covariates. Assuming a small effect (Cohen's f^2^ = 0.025), α = 0.05, and 80% power, the required sample size was approximately 510 using G^*^Power 3.1; allowing 10% non-completion, the target was 565. In practice, 600 women were surveyed across 10 Community Health Service Centers; after applying eligibility criteria and data-quality checks, 585 participants were included in the final analyses.

### 2.6 Measures

#### 2.6.1 Outcome: help-seeking intention

Help-seeking intention was assessed with a single item: “*Are you likely to seek medical help for urinary incontinence within the next six months?”* Responses used a five-point Likert scale from 1 (“definitely not”) to 5 (“definitely yes”). The variable was treated as ordinal in the primary analyses.

#### 2.6.2 IMB model constructs information

**SUI knowledge** was assessed with a 20-item questionnaire covering daily management, disease knowledge, and pelvic floor/functional exercise techniques. Items were scored dichotomously (correct = 1; incorrect/unknown = 0), and summed to yield a total of 0–20, with higher scores indicating greater knowledge. Internal consistency was good (Cronbach's α = 0.881).

#### 2.6.3 Motivation – personal

**Urinary incontinence–related life burden:** The 22-item Incontinence Quality of Life questionnaire [I-QOL; subscales: avoidance/limiting behaviors (8 items), psychosocial impact (9 items), social embarrassment (5 items)] (23) was administered with five response options per item ranging from “extremely” to “not at all.” In the original scoring system, higher scores indicate better quality of life. To facilitate interpretation and ensure directional consistency across measures, all items were reverse-scored (new = 6 – original) so that higher values indicate worse quality of life/greater symptom burden. The item mean (range 1–5) served as the primary score (Cronbach's α = 0.980).

**SUI Stigma:** The revised Stigma Impact Scale (SIS, 20 items) was used (e.g., “*I feel that others avoid me because of my condition*”). Items were rated 1–4 (strongly disagree to strongly agree) and summed/averaged; higher scores indicate stronger stigma (Cronbach's α= 0.970).

#### 2.6.4 Motivation – social

**Subjective Norms: Three** investigator-developed items asked how family, friends, and colleagues would view seeking care for SUI (1 = strongly oppose to 5 = strongly support). Higher scores denote stronger supportive norms (Cronbach's α= 0.716).

**Patient–Provider Communication:** The 15-item Communication Assessment Tool (CAT) (24) captured patients' communication experiences with healthcare providers on a five-point scale; higher scores reflect better communication (Cronbach's α = 0.958).

#### 2.6.5 Behavioral skills

**Self-efficacy for care-seeking:** We assessed core skills relevant to seeking professional care for SUI using three adapted items that capture both capability and confidence: (1) “*I can decide promptly to seek medical care when experiencing physical discomfort*,” (2) “*I can effectively cooperate with healthcare providers during treatment* (e.g., follow instructions),” and (3) “*I can clearly and succinctly describe my symptoms to the doctor*.” Items were rated on a five-point Likert scale (1 = never to 5 = always); higher scores indicate stronger self-efficacy (Cronbach's α = 0.905).

#### 2.6.6 Demographic and biomedical characteristics

Sociodemographic variables included age, education (primary or below / junior middle / senior high or above), marital status (married vs. non-married), monthly household income ( ≤ 5,000; 5,001–9,999; ≥10,000 CNY), medical insurance type (urban employee vs. urban/rural resident), pre-retirement occupation (manual / service / clerical / professional or administrative), co-residence (with spouse/children vs. living alone), and family doctor contract (yes/no). Biomedical variables included body mass index (BMI), number of chronic conditions (none/one/two/≥3), and urinary incontinence severity by the ICIQ-UI Short Form (ICIQ-UI SF) (25). The ICIQ-UI SF comprises three scored items—frequency, amount of leakage, and overall interference (total 0–21)—plus one unscored diagnostic item describing leakage circumstances. Total scores were categorized as mild (0–7), moderate (8–13), and severe (14–21).

### 2.7 Statistical analysis

Descriptive statistics summarized participant characteristics as mean ± standard deviation (SD) for continuous variables and n (%) for categorical variables. Between-group comparisons by help-seeking intention (≥4 vs. < 4) used independent-samples *t* tests and χ^2^ tests. Scale scores from the IMB framework were treated as continuous variables; for regression analyses, all continuous predictors were z-standardized so that effects reflect a one-SD change.

Latent class analysis (LCA) was conducted to identify socioeconomic subgroups based on education, income, health insurance type, and pre-retirement occupation, with model fit evaluated by AIC and BIC.

The main analysis employed an ordered logistic regression model to examine associations between the constructs of the IMB framework and help-seeking intention, adjusting for socioeconomic class and relevant covariates (age, marital status, living arrangement, gravidity, chronic conditions, incontinence severity, and family doctor contract). The proportional odds assumption was tested using the Brant test. Given evidence of an overall deviation, a partial proportional odds (PPO) model allowing incontinence severity (only) to vary across thresholds was fitted as a robustness check. Multicollinearity was assessed by generalized variance inflation factors (VIFs). A series of nested models was fitted to estimate the attenuation of socioeconomic effects after sequential inclusion of IMB components. Model performance was evaluated using AIC and McFadden's pseudo-R^2^. Potential non-linear associations for key IMB constructs (stigma, subjective norms) were tested by adding quadratic terms and comparing models using likelihood-ratio tests. Finally, sensitivity analyses using binary logistic regression (high intention ≥4 vs. < 4) and a restricted sample of women aged ≥60 years were performed to assess the robustness of findings. An exploratory Spearman correlation analysis among IMB-related constructs and help-seeking intention was additionally conducted to describe the relationships between psychosocial variables.

All analyses were performed in R (version 4.2.1) with a two-tailed significance level of 0.05.

## 3 Results

### 3.1 Participant characteristics

A total of 585 women aged 50 years and older were included in the analysis (mean age = 71.1 ± 9.3 years). Table 1 summarizes sample characteristics by help-seeking intention (≥4 vs. < 4). Two hundred and twenty three participants (38.1%) reported high help-seeking intention (scores ≥ 4) and 362 (61.9%) reported low/moderate intention (scores < 4). Most participants were married (69.1%) and living with family members (74.0%); 40.3% had signed a family-doctor contract. Based on latent class analysis combining education, income, insurance type, and pre-retirement occupation, 27.9% were categorized as high SES.

**Table 1:** Participant characteristics by help-seeking intention (≥4 vs. < 4).

Characteristic	Overall (*N* = 585)	High intention (*N* = 223)	Low/Moderate intention (*N* = 362)	χ^2^/t	*p*-value
Sociodemographic					
Age	71.15 ± 9.25	70.74 ± 9.94	71.41 ± 9.51	0.840	0.401
BMI	24.12 ± 3.69	24.41 ± 3.77	23.95 ± 3.63	−1.486	0.138
Marital status					
Married	404 (69.1)	164 (73.5)	240 (66.3)	3.89	0.066
Unmarried/Divorced/Widowed	181 (30.9)	59 (26.5)	122 (33.7)		
Living with family					
Yes	433 (74.0)	175 (78.5)	258 (71.3)	3.724	0.054
No	152 (26.0)	48 (21.5)	104 (28.7)		
Family-doctor contract					
Yes	236 (40.3)	103 (46.2)	133 (36.7)	5.12	**0.024**
No	349 (59.7)	120 (53.8)	229 (63.3)		
SES				11.507	0.001
Low SES	422 (72.1%)	143 (64.1%)	279 (77.1%)		
High SES	163 (27.9%)	80 (35.9%)	83 (22.9%)		
Biomedical					
Gravidity				5.474	0.140
0	20 (3.4)	3 (1.3)	17 (4.7)		
1	246 (42.1)	95 (42.6)	151 (41.7)		
2	226 (38.6)	92 (41.3)	134 (37.0)		
≥3	93 (15.9)	33 (14.8)	60 (16.6)		
Chronic diseases					
0	131 (22.4)	51 (22.9)	80 (22.1)	4.32	0.229
1	179 (30.6)	59 (26.5)	120 (33.1)		
2	135 (23.1)	51 (22.9)	84 (23.2)		
≥3	140 (23.9)	62 (27.8)	78 (21.5)		
SUI severity				52.55	< 0.001
Mild	341 (58.3)	88 (39.5)	253 (69.9)		
Moderate	215 (36.8)	119 (53.4)	96 (26.5)		
Severe	29 (5.0)	16 (7.2)	13 (3.6)		
IMB constructs					
SUI knowledge (0–20)	18.36 ± 4.37	17.11 ± 4.92	19.03 ± 3.76	5.31	**< 0.001**
Urinary incontinence–related life burden (1–5)	2.07 ± 0.71	2.39 ± 0.67	1.85 ± 0.69	−9.43	**< 0.001**
SUI Stigma (1–5)	2.88 ± 0.60	3.16 ± 0.64	2.71 ± 0.50	−9.37	**< 0.001**
Subjective Norms (1–5)	3.00 ± 0.55	3.36 ± 0.60	2.80 ± 0.49	−11.76	**< 0.001**
Patient–Provider Communication (1–5)	3.59 ± 0.57	3.73 ± 0.50	3.51 ± 0.61	−4.22	**< 0.001**
Care-seeking self-efficacy (1–5)	3.43 ± 0.69	3.66 ± 0.64	3.30 ± 0.77	−5.89	**< 0.001**

Values are presented as mean±standard deviation (SD) or *n* (%). Significant results are in bold (*P* < 0.05).

Regarding clinical and psychosocial characteristics, 15.9% had three or more pregnancies, and 41.8% reported moderate to severe UI. Mean (SD) scores on IMB constructs were: knowledge 18.36 (4.37), life burden 2.07 (0.71), stigma 2.88 (0.60), subjective norms 3.00 (0.55), patient–provider communication 3.59 (0.57), and self-efficacy 3.43 (0.69).

Women with higher help-seeking intention scored significantly higher on life-burden, stigma, subjective norms, patient–provider communication, and self-efficacy, but lower on knowledge (*p* < 0.001 for all). They were also more likely to have a family-doctor contract, whereas age, SES, and clinical factors did not differ significantly between groups.

### 3.2 Latent class analysis of socioeconomic status

Latent class analysis based on education, household income, medical insurance, and pre-retirement occupation identified a three-class solution as optimal (BIC = 3,825.3; AIC = 3,711.6; LogLik = −1,829.8). Classification quality was acceptable (average posterior probabilities 0.812–0.913; classification error 0.136).

Class 1 (35.1%) comprised women with predominantly low education and income, resident medical insurance, and non-technical or manual work—representing a low socioeconomic group with limited formal employment. Class 2 (38.2%) was characterized by middle education and income, near-universal urban employee insurance, and mainly manual occupations—reflecting a mid-level SES group with stable but labor-intensive employment. Class 3 (26.7%) included highly educated women with middle-to-high income, professional or administrative jobs, and almost universal urban employee insurance—typifying a high SES group with strong educational and occupational advantages.

For subsequent analyses, Classes 1 and 2 were merged as Low SES, and Class 3 was defined as High SES. Full conditional probabilities are provided in Appendix Table S1.

### 3.3 IMB-based predictors of help-seeking intention

Variance inflation factors (VIFs) ranged from 1.15 to 4.60 (mean = 1.64), indicating no evidence of multicollinearity among independent variables (Appendix Table S2).

In the full proportional-odds model (Table 2), higher stigma (cOR = 1.80, 95% CI 1.35–2.40), stronger subjective norms (cOR = 2.81, 95% CI 2.25–3.51), and better patient–provider communication (cOR = 1.27, 95% CI 1.04–1.54) were significantly associated with higher help-seeking intention. Greater life burden (cOR = 1.25, 95% CI 0.91–1.72) and self-efficacy (cOR = 1.16, 95% CI 0.95–1.43) showed positive but non-significant trends, whereas higher knowledge (cOR = 0.81, 95% CI 0.67–0.97) was inversely associated with intention.

**Table 2:** Ordered logistic regression of help-seeking intention.

Variable	cOR	95% CI	*p* value
Socioeconomic status: High vs. Low	1.16	0.78–1.72	0.46
Information			
Knowledge (z)	0.81	0.67–0.97	**0.02**
Motivation — personal			
Stigma (z)	1.80	1.35–2.40	**< 0.001**
Life burden (z)	1.25	0.91–1.72	0.15
Motivation — social			
Subjective norms (z)	2.81	2.25–3.51	**< 0.001**
Patient–provider communication (z)	1.27	1.04–1.54	**0.02**
Behavioral skills			
Care-seeking self-efficacy (z)	1.16	0.95–1.43	0.13

All models adjusted for age, marital status, living arrangement, gravidity, chronic conditions, symptom severity, and family doctor contract. cOR, cumulative odds ratio; CI, confidence interval; all continuous variables standardized (z-scores). Bold values indicate statistical significance at *P* < 0.05.

Among covariates, having a family doctor contract was positively associated with help-seeking intention (cOR = 2.10, 95% CI 1.47–3.00). Age showed a modest negative association (cOR = 0.82, 95% CI 0.68–1.00). Other factors—including marital status, living arrangement, parity, chronic conditions, and symptom severity—were not statistically significant.

**Figure 2:**
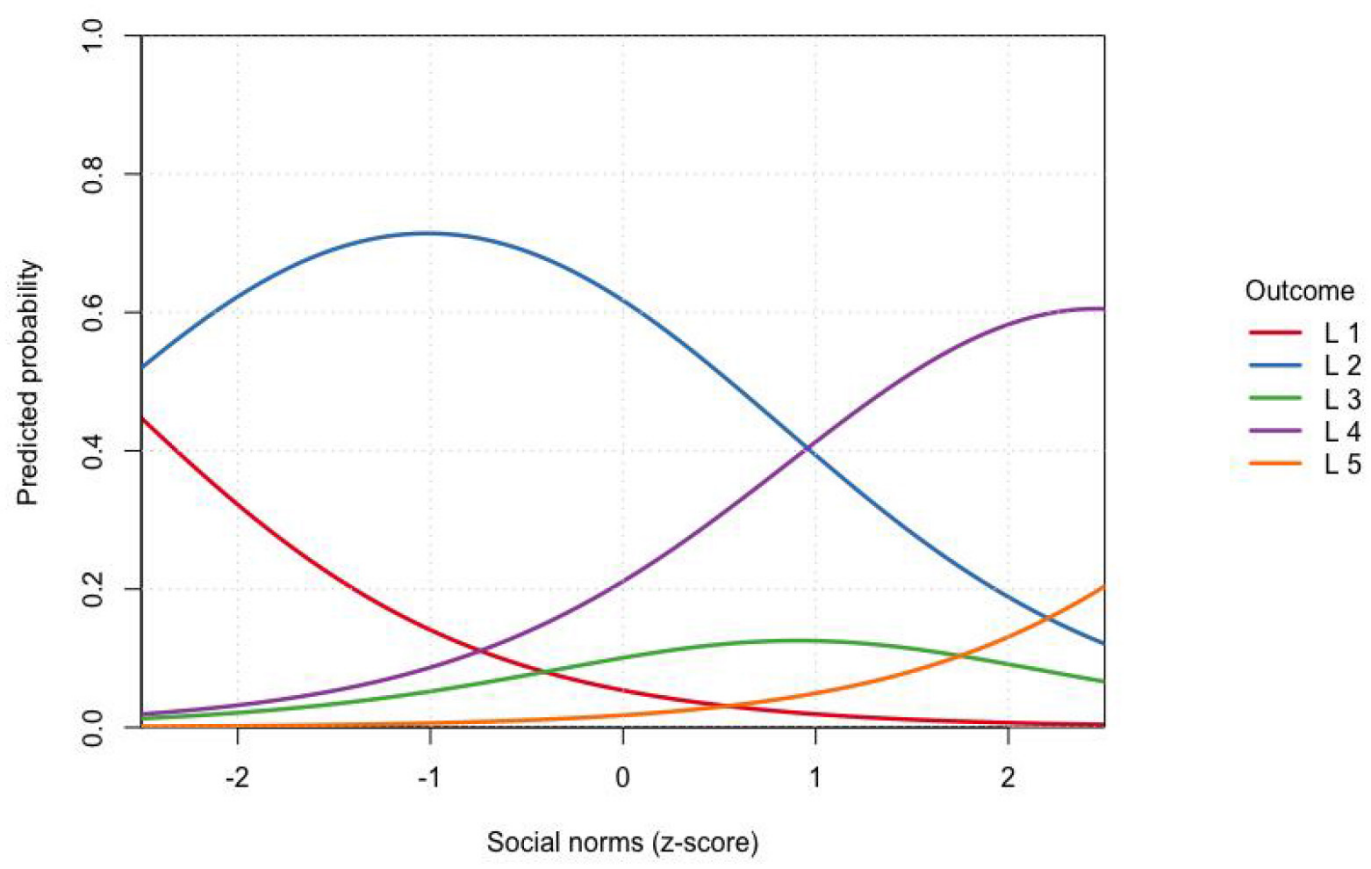
Predicted probabilities of help-seeking intention across levels of social norms.

Figure 2 illustrates the predicted probabilities of help-seeking intention across the range of subjective norms. A clear monotonic pattern was observed: as perceived social support for seeking medical care increased, the probabilities of higher intention levels (scores 4–5) rose sharply, while the probabilities of low intention (scores 1–2) declined. This visualization underscores the strong and graded influence of social motivation on care-seeking intention.

Quadratic terms for stigma and subjective norms were tested in the ordered logistic model [Δχ^2^(2) = 3.04, *p* = 0.22], showing no evidence of non-linearity (Appendix Table S3).

### 3.4 SES-related attenuation and model comparison

Sequential models incorporating IMB components demonstrated progressive attenuation of the SES effect on help-seeking intention (Table 3). In the baseline model (M0: SES + covariates), women with high SES were more likely to report higher intention (cOR = 1.90, 95% CI 1.34–2.69). After adding information (M1), the association attenuated by 23.5% (cOR = 1.63, 95% CI 1.14–2.34). Inclusion of motivational factors (M2)—further reduced the SES effect by 77.0% (cOR = 1.16, 95% CI 0.79–1.71). When behavioral skills were added (M3), the SES coefficient became non-significant (cOR = 1.12, 95% CI 0.76–1.66), reflecting an overall attenuation of 82.2%.

**Table 3:** Stepwise models showing attenuation of the SES coefficient (M0–M3).

Model (block specification)	SES (High vs. Low) cOR (95% CI)	AIC	McFadden's R^2^	Attenuation vs. M0 (%)
M0: SES + covariates	1.90 (1.34–2.69)	1,486	0.053	—
M1: M0 + Information (Knowledge)	1.63 (1.14–2.34)	1,473	0.062	23.5
M2: M1 + Motivation (Stigma; Norms)	1.16 (0.79–1.71)	1,282	0.193	77.0
M3: M2 + Behavioral skills	1.12 (0.76–1.66)	1,279	0.195	82.2

All models are proportional-odds logistic regressions with continuous predictors z-standardized (per 1 SD).

Model fit improved consistently (AIC: 1486 → 1279; McFadden's R^2^: 0.05 → 0.20), supporting the IMB framework's explanatory value in mediating the SES–intention relationship.

### 3.5 Sensitivity analyses

The proportional-odds assumption was evaluated using the Brant test. The global Brant test indicated an overall deviation from proportional odds (χ^2^ = 42.1, *p* < 0.001). To assess the robustness of the primary inferences to this violation, we fitted a PPO model that relaxed the proportional-odds constraint for symptom severity (nominal = severity). The PPO model provided a significantly improved fit compared with the proportional-odds model (likelihood ratio test: LR χ^2^ = 26.586, df = 6, *p* = 0.00017), while the key IMB predictors retained the same direction and similar magnitude of associations. Given the interpretability and parsimony of the ordered specification, and the stability of the key IMB effects under PPO, we retained the proportional-odds framework for the primary analyses (Appendix Table S4).

To assess the robustness of the main findings, a sensitivity analysis was performed using a dichotomized outcome of help-seeking intention (high intention ≥ 4 vs. < 4) (Appendix Table S5). The direction and magnitude of the associations were consistent with the ordered logistic model. Participants with higher subjective norms (OR = 2.68, 95% CI = 2.01–3.57, *p* < 0.001) and greater perceived stigma (OR = 2.07, 95% CI = 1.41–3.05, *p* < 0.001) were significantly more likely to report high intention to seek care. Self-efficacy showed a positive but marginal association with high intention (OR = 1.28, 95% CI = 0.98–1.68, *p* = 0.066), whereas patient–provider communication was positively associated with high intention (OR = 1.30, 95% CI = 1.01–1.67, *p* = 0.042). Knowledge scores were inversely associated with help-seeking (OR = 0.74, 95% CI = 0.58–0.94, *p* = 0.012). In addition, to address the definition of “older adults” in China, we conducted a sensitivity analysis restricted to women aged ≥ 60 years. In this subsample, key IMB associations remained materially unchanged, and the stepwise attenuation of the SES–intention association was comparable to the primary analysis (Appendix Table S6).

Finally, an exploratory Spearman correlation analysis among IMB-related constructs and help-seeking intention is presented in Appendix Table S7. Help-seeking intention was positively correlated with life burden (*r* = 0.361), stigma (*r* = 0.362), subjective norms (*r* = 0.455), patient–provider communication (*r* = 0.185), and care-seeking self-efficacy (*r* = 0.237), while information showed a negative correlation (*r* = −0.181) (all *p* < 0.001). These correlations are descriptive and are reported to contextualize the clustering of IMB constructs rather than to imply causal relationships.

## 4 Discussion

This study examined how informational, motivational, and behavioral factors jointly shape older women's intention to seek care for SUI in urban China. Guided by the IMB model, we found that subjective norms and stigma—rather than SES—were the dominant predictors of help-seeking intention. Once information, motivational and skill-related constructs were accounted for, the direct association between SES and intention was substantially attenuated, suggesting that psychosocial mechanisms may bridge or buffer structural inequalities in care-seeking behavior.

In this study, only 38.1% of women reported an intention to seek medical care for SUI within the next six months, a proportion notably lower than the 65.4% positive attitude toward care-seeking reported in a recent large-scale population-based survey of 54,346 Chinese women (9). Several factors may account for this discrepancy. First, the prior study assessed general attitudes toward seeking care, whereas our measure captured a time-bound behavioral intention among women with questionnaire-identified symptoms, a more stringent criterion. Second, our participants were older on average, and help-seeking intention tends to decline with age due to mobility constraints, symptom normalization, and perceived treatment futility (26). Third, contextual differences matter: while the urban Shanghai setting offers dense service networks and high health literacy, intention remained strongly patterned by SES, symptom severity, education, and engagement with family doctors (27). These findings suggest that favorable attitudes alone may not translate into concrete intention, highlighting the importance of proximal psychosocial drivers—particularly subjective norms and stigma—and the local service ecology that enables or inhibits action.

The salience of subjective norms highlights the social nature of health decision-making in later life and within collectivist settings. Older women who perceived stronger approval or encouragement from family and peers were markedly more likely to report care-seeking intention. This finding echoes prior qualitative and quantitative research suggesting that social expectations in family-centered networks act as both enablers and gatekeepers of care (14, 28). In this normative landscape, informational campaigns alone may have limited traction; interventions should aim to shift perceived community expectations by mobilizing influential peers, family members, and local organizations. Neighborhood infrastructures—such as residents' committees, senior associations, and WeChat-based health networks—can amplify new norms through repeated interpersonal exposure, transforming help-seeking from a private decision into a socially endorsed practice.

The positive association between stigma and intention diverges from conventional assumptions but aligns with several Chinese studies where higher perceived stigma coincided with greater help-seeking readiness (29). One plausible interpretation is that stigma co-varies with symptom salience and distress—as perceived threat and secrecy burdens rise, so does the motivation to seek relief. This may reflect a threshold or “problem activation” mechanism rather than a linear deterrent effect. Although formal tests of non-linearity were not significant, a weak quadratic signal for norms suggests that the interplay between cognitive (belief-based) and affective (shame-related) components merits further exploration. Future research should disentangle these dimensions to clarify when stigma acts as a barrier vs. a catalyst for action.

Knowledge showed weak and inconsistent associations with intention, reinforcing that information alone is insufficient to prompt help-seeking unless embedded within supportive social and motivational contexts. This aligns with evidence that knowledge primarily functions as an enabling rather than driving determinant once stigma and norms are addressed (15, 30). Similarly, both patient–provider communication and self-efficacy for care-seeking were directionally positive but not statistically significant. These findings imply that enhancing communication skills or confidence may only translate into behavior after normative constraints are relaxed and help-seeking becomes socially legitimate. Targeted training for community clinicians to initiate sensitive discussions, invite disclosure, and respond empathically could thus facilitate the transition from intention to action.

The attenuation of SES effects after introducing IMB constructs suggests that psychosocial resources partially mediate SES disparities in care-seeking. In contexts where structural barriers (e.g., cost, accessibility) are relatively low—as in metropolitan Shanghai—social-cognitive mechanisms may exert stronger proximal influence than economic resources (31). This finding complements broader literature on “psychosocial equalization,” which posits that cohesive networks and accessible community infrastructure can mitigate the behavioral expression of SES inequality (32). Strengthening these psychosocial pathways could therefore represent a cost-effective lever for reducing inequities in preventive and rehabilitative service use among older women.

These results highlight the need for multi-level interventions that move beyond information dissemination toward norm transformation and stigma reduction. Community-based peer campaigns, storytelling via social media, and inclusion of continence management in family-doctor services may collectively normalize help-seeking. At the professional level, training community physicians to routinely ask about urinary symptoms and to frame help-seeking as normative and manageable could further close the gap between intention and action. Given China's rapidly aging population and the ongoing expansion of primary care networks, embedding such approaches within existing health-promotion infrastructures is both feasible and scalable.

Several limitations warrant consideration. First, the cross-sectional design limits causal inference, and we assessed intention rather than realized care utilization. Second, the sample was drawn from an urban district with relatively high service density, limiting generalizability to rural or underserved areas. Third, although the proportional-odds assumption was violated overall, key IMB predictors (norms, stigma) satisfied item-level checks, and findings were robust in sensitivity analyses using binary outcomes. Fourth, self-efficacy for care-seeking measures were generic and may not capture SUI-specific behavioral competencies; refinement and contextual validation are needed. Fifth, SES classification via latent class analysis and subsequent dichotomization may obscure within-group heterogeneity and underestimate uncertainty. Finally, reliance on self-report introduces potential common-method bias and social desirability effects; longitudinal or mixed-method designs could mitigate these concerns and deepen understanding of the normative and emotional dynamics underlying SUI help-seeking.

## 5 Conclusions

In sum, this study demonstrates that subjective norms outweigh SES in shaping help-seeking intention for stress urinary incontinence among older urban women. The findings underscore the need to reframe help-seeking as a socially supported, normative act rather than an individual admission of failure. By integrating psychosocial insight into primary care outreach and community health programs, interventions can more effectively transform awareness into action, reducing the unmet burden of incontinence in aging populations.

## Data Availability

The datasets generated and/or analyzed during the current study are not publicly available due to participant confidentiality and the sensitive nature of the data, but are available from the corresponding author on reasonable request.
