# Supplementary material for: Socioeconomic differences in help-seeking intention for stress urinary incontinence: evidence from an information–motivation–behavioral skills model framework
Source: Front Public Health. 2026 Apr 29;14:1794464. doi: 10.3389/fpubh.2026.1794464 (PMC13168019; doi:10.3389/fpubh.2026.1794464)
Supplement: Supplementary file 1 [file Supplementary_file_1.pdf]

The 3-class solution demonstrated the lowest BIC and acceptable entropy (0.65), supporting its selection as the optimal model. Average posterior probabilities (AvePP) indicated good classification reliability.

Table S1. Fit statistics and classification quality for 2–4 class LCA models of SES

Classes	Log-likelihood	AIC	BIC	Entropy	Classification error	AvePP (range)
2	−1875.275	3784.551	3858.868	—	—	—
3	−1829.823	3711.645	3825.307	0.65	0.14	0.81–0.91
4	−1822.879	3715.758	3868.765	—	—	—

Adding quadratic terms did not significantly improve model fit, though the borderline signal for subjective norms (p = 0.082) suggests possible threshold effects warranting further study.

Table S2. Variance inflation factors (VIFs) for variables in the proportional-odds model

Variable	GVIF	df	Adjusted GVIF^(1/(2*df))
SES	1.17	1	1.08
knowledge	1.26	1	1.12
Urinary incontinence – related life burden	3.77	1	1.94
stigma	3.03	1	1.74
norms	1.46	1	1.21
Subjective Norms	1.53	1	1.24
Patient–Provider Communication	1.39	1	1.18
age	1.40	1	1.18
Marital Status	4.60	1	2.14
Living with family	4.50	1	2.12
Gravidity	1.25	3	1.04
Chronic Diseases	1.29	3	1.04
SUI severity	2.51	2	1.26
Family-doctor contract	1.15	1	1.07

Adding quadratic terms did not significantly improve model fit. A borderline effect for subjective norms ($p = 0.082$) may suggest threshold or diminishing-return patterns worth exploring in future research.

Table S3 Nonlinear (quadratic) term tests for stigma and subjective norms

Test	$\Delta\chi^2$	df	p
Add both quadratic terms	3.04	2	0.218
Drop subjective norms^2	3.02	1	0.082
Drop stigma^2	0.008	1	0.931

Table S4 Partial proportional odds model (PPO) as a robustness check for the proportional-odds assumption

Predictor	OR (95% CI)	p value
SES (High vs Low)	1.20 (0.80–1.79)	0.372
Family doctor contract (yes vs no)	2.16 (1.50–3.10)	<0.001
Information / knowledge (z)	0.79 (0.66–0.95)	0.015
Stigma (z)	1.86 (1.39–2.49)	<0.001
Subjective norms (z)	2.85 (2.27–3.57)	<0.001
Patient–provider communication (z)	1.29 (1.06–1.58)	0.011
Life burden (z)	1.27 (0.92–1.75)	0.155
Care-seeking self-efficacy (z)	1.17 (0.95–1.43)	0.137

Severity was specified as a nominal effect, allowing its association with intention to vary across thresholds; threshold-specific parameters are omitted for brevity.

Table S5 Sensitivity analysis using binary logistic regression (High intention $\geqslant$ 4 vs $<$ 4)

**Variable**	**OR**	**95% CI**	**p-value**
SES (High vs Low)	1.53	0.93 – 2.52	0.074
Knowledge (z)	0.74	0.58 – 0.94	0.012
Life burden (z)	1.37	0.90 – 2.09	0.145
Stigma (z)	2.07	1.41 – 3.05	<0.001
Social norms (z)	2.68	2.01 – 3.57	<0.001
Self-efficacy (z)	1.28	0.98 – 1.68	0.066
Patient–provider communication (z)	1.30	1.01 – 1.67	0.042

Table S6 Sensitivity analysis restricted to women aged ≥60 years (stepwise ordered logistic regression, M0–M3)

Model	Predictor	OR (95% CI)	p
M0	SES (High vs Low)	**1.93 (1.33–2.79)**	**<0.001**
M1	SES (High vs Low)	**1.65 (1.13–2.41)**	**0.010**
M1	Information (z)	**0.68 (0.57–0.82)**	**<0.001**
M2	SES (High vs Low)	1.17 (0.78–1.77)	0.439
M2	Information (z)	**0.76 (0.63–0.92)**	**0.005**
M2	Stigma (z)	**1.96 (1.43–2.69)**	**<0.001**
M2	Subjective norms (z)	**3.00 (2.38–3.78)**	**<0.001**
M2	Patient–provider communication (z)	**1.29 (1.06–1.57)**	**0.012**
M2	Life burden (z)	1.02 (0.72–1.45)	0.905
M3	SES (High vs Low)	1.14 (0.76–1.73)	0.520
M3	Information (z)	**0.78 (0.64–0.95)**	**0.012**
M3	Stigma (z)	**1.99 (1.46–2.73)**	**<0.001**
M3	Subjective norms (z)	**2.91 (2.29–3.69)**	**<0.001**
M3	Patient–provider communication (z)	**1.25 (1.02–1.54)**	**0.030**
M3	Life burden (z)	1.03 (0.72–1.46)	0.886
M3	Care-seeking self-efficacy (z)	1.13 (0.91–1.41)	0.264

Models M0–M3 follow the same stepwise specification as the primary analysis. IMB constructs were standardized (z-scores). ORs are cumulative odds ratios from ordered logistic regression.

Table S7 Spearman correlations among IMB-related constructs and help-seeking intention

Variable	Knowledge	Life burden	Stigma	Subjective norms	Patient–provider communication	Care-seeking self-efficacy	Help-seeking intention
**Knowledge**	1						
**Life burden**	-0.026	1					
**Stigma**	0.099*	0.784**	1				
**Subjective norms**	-0.251**	0.160**	0.151**	1			
**Patient–provider communication**	-0.260**	0.001	-0.102*	0.334**	1		
**Care-seeking self-efficacy**	-0.377**	0.007	-0.073	0.404**	0.428**	1	
**Help-seeking intention**	-0.182**	0.367**	0.373**	0.529**	0.208**	0.255**	1

* $p < 0.05$

** $p < 0.01$
